# O-GlcNAcylation in the osteoblast lineage—boosting the complexity of Wnt-stimulated bone formation

**DOI:** 10.1038/s44319-024-00242-2

**Published:** 2024-09-10

**Authors:** Sandra Pohl, Thorsten Schinke

**Affiliations:** Department of Osteology and Biomechanics, University Medical Center Hamburg-Eppendorf, 20246 Hamburg, Germany

**Keywords:** Development, Post-translational Modifications & Proteolysis, Signal Transduction

## Abstract

The molecular mechanisms explaining the osteogenic influence of Wnt molecules are still not fully clarified. A study in this issue shows that O-GlcNAcylation is required for the osteoanabolic effects of Wnt stimulation.

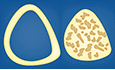

## Teaser

Although differentiation and activity of bone-forming osteoblasts are regulated at various levels, there is ample evidence that components of the Wnt signaling pathway play a dominant role in bone mass acquisition. Moreover, a neutralizing antibody blocking the Wnt signaling inhibitor sclerostin has recently been established as a novel osteoanabolic treatment option for osteoporotic patients. Importantly, however, the molecular mechanisms explaining the osteogenic influence of Wnt molecules are still not fully clarified. In this issue of *EMBO Reports*, You and co-workers show that O-GlcNAcylation is required for the osteoanabolic effect of Wnt stimulation, in vitro and in vivo (You et al, 2024).

Bone is a highly dynamic tissue undergoing lifelong remodeling, which is mediated by bone-forming osteoblasts and bone-resorbing osteoclasts. Importantly, osteoblasts and osteoclasts are fundamentally different cell types, which differ in terms of progenitors, morphology, mode of action, and regulatory molecules. This explains why current treatment options to counteract bone loss in osteoporotic patients are either targeting the osteoclast population (anti-resorptive treatment) or aiming at stimulating osteoblast activity (osteoanabolic treatment). Since intact bone remodeling, and thereby the constant renewal of aged and defective bone matrix, is a prerequisite for long-term skeletal integrity, osteoanabolic treatment options are principally favorable (Rachner et al, 2011). Importantly, although the vast majority of osteoporotic patients are currently still treated with anti-resorptive drugs, there are new developments in terms of osteoanabolic treatment, initially based on human genetic findings.

More specifically, the genetic analysis of patients with osteosclerotic disorders, i.e., high bone mass due to activation of matrix-producing osteoblasts, has identified pathogenic variants in two skeletal disease genes, i.e., *LRP5* (gain-of-function) and *SOST* (loss-of-function), both linked to activated Wnt signaling (Huybrechts et al, 2020). Whereas LRP5 was already established as a co-receptor for Wnt ligands, the *SOST-*encoded protein sclerostin was found to be specifically secreted by terminally differentiated osteoblasts and to bind to the extracellular region of LRP5 thereby inhibiting Wnt interaction. Based on these relevant discoveries, it was possible to develop an effective osteoanabolic medication, i.e., a monoclonal antibody (Romosozumab) neutralizing the inhibitory effect of sclerostin (Tanaka and Matsumoto, 2021). In this context, it is further important to refer to WNT1, a potent osteoanabolic molecule, for which monoallelic or biallelic variants were found to cause early-onset osteoporosis or osteogenesis imperfecta, respectively (Luther et al, 2018). Moreover, there is also evidence for an influence of rare variants in other *WNT* genes on bone mineral density, one of them being *WNT3A*.

In contrast to the strong in vivo evidence for a key role of Wnt signaling in bone formation, which was also supported in respective model systems (such as genetically modified mouse lines), the intracellular pathways explaining the osteoanabolic action of Wnt ligands are still not fully clarified. Moreover, since most Wnt ligands promote a signaling pathway involving the stabilization of ß-catenin (canonical Wnt signaling), it came as a surprise that mice with an osteoblast-specific inactivation of ß-catenin primarily displayed increased bone resorption, unlike mice lacking *Lrp5* or *Sost*, where the bone formation rate was most strongly affected (Baron and Kneissel, 2013). That non-canonical signaling pathways could be more relevant in osteoblasts was also supported by other studies, for instance, regarding the function of Wnt1, for which mTORC1 activation was found to be involved in enhancing osteogenic differentiation of the mesenchymal cell line ST2 (Joeng et al, 2017).

**Figure 1:**
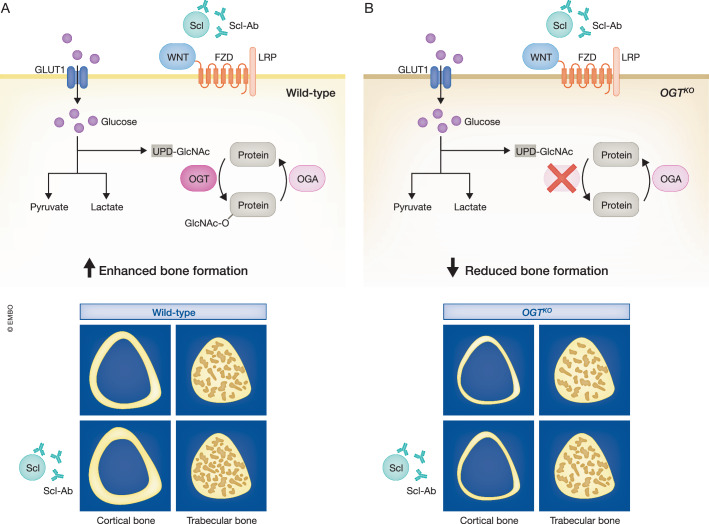
Requirement of OGT for Wnt-induced bone formation in vivo. (**A**) In the presence of OGT, UDP-GlcNAc is utilized for O-GlcNAcylation of target proteins, which is activated by WNT binding to a Frizzled (FZD) receptor and a co-receptor (LRP5 or LRP6). Injection of an anti-sclerostin antibody (Scl-Ab) activates Wnt signaling, thereby enhancing bone formation in the cortical and trabecular bone compartment. (**B**) Inactivation of OGT in osteoblast lineage cells does not only result in reduced bone formation in the absence of Scl-Ab but also impairs the response towards Scl-Ab specifically in the cortical bone compartment.

In this issue of *EMBO Reports*, You et al, also utilize ST2 cells in order to study the molecular influence of Wnt3a on osteogenesis (You et al, 2024). They thereby identify another potential mechanism by which Wnt signaling promotes bone formation, involving O-GlcNAcylation of protein substrates. Similar to phosphorylation events, but far less established until now, O-GlcNAcylation is a dynamic and reversible posttranslational modification affecting specific cellular processes (Zeidan and Hart, 2010). The two critical enzymes in this regard are O-GlcNAc transferase (OGT), which modifies serine or threonine residues of target proteins by a single O-linked ß-N-acetylglucosamine residue, and O-GlcNAc hydrolase (OGA), which removes the respective modification (Yang and Xian, 2017). Since OGT activity depends on the presence of UDP-GlcNAc, the end product of the hexosamine biosynthetic pathway, the process of O-GlcNAcylation is also linked to glucose metabolism (Fig. 1), which is particularly relevant in osteoblasts.

The manuscript by You et al, starts with an unbiased screening approach for metabolites affected by Wnt3a treatment of ST2 cells. The authors thereby identified an increase of UDP-GlcNAc, which led to many additional experiments, including the demonstration that Wnt3a increases the expression of proteins required for O-GlcNAcylation: OGT and glutamine fructose 6-phosphate amidotransferase 1 (GFAT1), the rate-limiting enzyme of the hexosamine biosynthetic pathway. Using a combination of inhibitor treatment and siRNA knockdown experiments, the authors show that O-GlcNAcylation is required for Wnt3a-induced osteoblastogenesis of ST2 cells, but also for their Wnt3a-stimulated glucose uptake and aerobic glycolysis (You et al, 2024). Through a remarkable amount of work, You et al, further succeed to discriminate a rapid induction of O-GlcNAcylation via Ca^2+^-induced PKA-mediated phosphorylation of GFAT1 from a prolonged stimulation depending on ß-catenin, i.e., canonical Wnt signaling. Finally, the authors identify pyruvate dehydrogenase kinase 1 (PDK1) being O-GlcNAcylated (at Ser^174^) in response to Wnt3a (You et al, 2024). The gatekeeper PDK1 regulates the conversion of the glucose metabolite pyruvate to acetyl-coA, which is then oxidized to produce energy via the citric acid cycle. The Wnt-induced O-GlcNAcylation of PDK1 stabilizes the protein and thereby increases the conversion of pyruvate to lactate (aerobic glycolysis). This reprogramming of glucose metabolism was shown to promote bone formation (Esen et al, 2013).

With respect to the above-mentioned clinical impact of this research area, it is particularly important that the authors also demonstrate the in vivo relevance of their findings. More specifically, they generated a mouse model allowing an inducible deletion of OGT in osteoblast lineage cells. These mice were used to inactivate OGT (by doxycycline removal from the drinking water), and their skeletal phenotyping performed 4 weeks later revealed reduced trabecular and cortical bone mass compared to the respective controls (You et al, 2024). Importantly, the authors also analyzed the response of these animals to Wnt activation achieved through repetitive injection of an anti-sclerostin antibody for a period of 4 weeks. Here they observed that the osteoanabolic influence of this treatment was fully abolished by the conditional inactivation of OGT in the cortical bone compartment, whereas the trabecular bone volume was significantly increased by anti-sclerostin antibody injection regardless of the OGT genotype (Fig. 1). In a second set of experiments the authors analyzed this mouse model in terms of bone fractures, which were surgically induced, in parallel to starting the OGT inactivation and/or anti-sclerostin antibody treatment (You et al, 2024). By analyzing the fracture callus in these animals, the authors found that conditional inactivation of OGT results in delayed fracture healing, although the response to anti-sclerostin antibody treatment was not fully impaired in these mice.

Collectively, these observations allow to draw at least two important conclusions regarding the in vivo relevance of O-GlcNAcylation in osteoblast lineage cells. First, OGT expression is required for physiological bone formation in both, the trabecular and cortical bone compartments. Second, the increase in cortical bone thickness achieved by Wnt signaling activation through anti-sclerostin antibody injection depends on the presence of OGT (Fig. 1). The findings reported by You et al, are not only novel and relevant, they also provide a basis for future studies regarding the impact of O-GlcNAcylation on bone remodeling. In fact, there are several questions that remain to be addressed. For instance, it is conceivable to speculate that PDK1 is not the only substrate, which is modified by O-GlcNAcylation after treatment by Wnt3a, and that additional regulatory mechanisms involving OGT might be relevant in osteoblast lineage cells. It would also be interesting to study if the molecular pathways identified by You et al, are specifically activated by Wnt3a, or if other osteoanabolic Wnt ligands, such as Wnt1, also act by promoting O-GlcNAcylation. Finally, it would be important to know if the observed influences of Wnt3a are specific for osteoblast lineage cells, or if similar events are activated to the same extent in any other cell type.
